# Integrating online and offline data for crisis management: Online geolocalized emotion, policy response, and local mobility during the COVID crisis

**DOI:** 10.1038/s41598-021-88010-3

**Published:** 2021-04-19

**Authors:** Shihui Feng, Alec Kirkley

**Affiliations:** 1 Unit of Human Communication, Development, and Information Sciences, Faculty of Education, The University of Hong Kong, Hong Kong, China; 2 Department of Physics, University of Michigan, Ann Arbor, Michigan USA

**Keywords:** Computational science, Information technology

## Abstract

Integrating online and offline data is critical for uncovering the interdependence between policy and public emotional and behavioral responses in order to aid the development of effective spatially targeted interventions during crises. As the COVID-19 pandemic began to sweep across the US it elicited a wide spectrum of responses, both online and offline, across the population. Here, we analyze around 13 million geotagged tweets in 49 cities across the US from the first few months of the pandemic to assess regional dependence in online sentiments with respect to a few major COVID-19 related topics, and how these sentiments correlate with policy development and human mobility. In this study, we observe universal trends in overall and topic-based sentiments across cities over the time period studied. We also find that this online geolocalized emotion is significantly impacted by key COVID-19 policy events. However, there is significant variation in the emotional responses to these policies across the cities studied. Online emotional responses are also found to be a good indicator for predicting offline local mobility, while the correlations between these emotional responses and local cases and deaths are relatively weak. Our findings point to a feedback loop between policy development, public emotional responses, and local mobility, as well as provide new insights for integrating online and offline data for crisis management.

## Introduction

An inherent challenge for integrating social media usage into crisis management is that most social-media based findings can help us understand the perspectives of online users regarding a crisis, but have limited capability to contribute to the development of on-site response strategies and relief activities due to a lack of connection with localized offline information. Social media data alone are not sufficient for assessing risks during emergency response scenarios, and bridging the gap between theoretical findings and practical applications of social media usage in crisis management requires integration of online and offline information. Large-scale user-generated social media data can be used for assessing public emotional responses to crises^1–3^, but connecting these online emotional responses with offline behavioral data (e.g. mobility data, purchase data) can provide more practical insight for predicting the localized situation during a crisis and developing effective and timely relief policy. In this study, we analyze the *online geolocalized emotion (OGE)—the sentiments derived from a set of geotagged content on social media*—towards COVID-19 using Twitter data. The relationships of OGE with COVID-related polices and offline mobility during this significant public health crisis are assessed, in an attempt to demonstrate the importance of utilizing online and offline data for uncovering the interdependence among public emotional responses, policy development, and human mobility in crisis management.

COVID-19, an infectious disease caused by a novel coronavirus, had infected around 3.2 million people around the world as of May 1st 2020, the end of the time period analyzed in this study, and has infected over 110 million people to date. This long-lasting and highly contagious epidemic has impacted every aspect of society, and is considered by many the greatest global challenge since World War II. In response to this crisis, beyond the enormous health care effort, scientific communities in various disciplines have been working together to better understand the economic, societal, and social effects of this crisis. As part of this effort, a number of studies have been conducted to examine the use of social media (e.g. Twitter, Weibo, and Facebook) during COVID-19. The topics of these studies mainly fall into two categories: assessing public mental health and feelings such as anxiety and fear towards COVID-19^4–10^, and diffusion of crisis-relevant and false information^11–16^. A few studies have utilized geotagged data on social media to map and predict the number of infected cases^17^ and offline mobility^18,19^. For instance, Huang et al.^19^ propose an approach to capture offline mobility in certain geographic regions through online geotagged data, in order to assess responsiveness to protection measures. In this study, we aim to examine online geolocalized emotion (OGE) toward COVID and explore its relationships with federal and local policy development as well as offline human mobility. The closest work we find to our study is that by Porcher and Renault^18^, who analyzed the relationship between the number of tweets relevant to social distancing and trends in human mobility at a state-level in the US using 402,005 tweets, finding through linear regression analysis that an increase in online discussion about social distancing is associated with a decrease in mobility with a one-day lag. Our study contributes to this line of discussion with a much larger dataset, examining online responses with geolocalized sentiments (rather than tweet counts) and using more comprehensive statistical methods to analyze the relationships between time series data to assess the connections between online responses and offline mobility, as well as the policy effects on OGE dynamics.

The research questions leading this study are: (1) What are the characteristics of OGE dynamics towards COVID-19 across US cities?; (2) How do federal and local policies affect OGE?; and (3) What are the relationships of OGE with offline mobility and infection data? Around 13 million geotagged tweets and daily city-level mobility data from February 26th to May 1st 2020 are used in this study to address these questions. Using measures derived from tweet sentiments, we quantify daily OGE regarding five key COVID-related subtopics across different locations, as well as its coherence across these locations. Using novel sentiment-based measures and tools from econometric time series analysis, in this study we find

1. There were strong universal OGE trends regarding COVID-19 and selected subtopics across the cities studied.
2. Despite the consistent overall trends, cities differed in the extent to which their OGE was influenced immediately following major federal and local policies.
3. OGE and mobility were correlated in many cities, while the association between epidemic measures and OGE was relatively weak or absent in most cities.
4. OGE can be used as an indicator for predicting mobility trends in cities.

This study provides an empirical demonstration of an effective analytic framework integrating social media data with offline data to examine the relationships between public emotional responses, policy development, and local mobility during crises. The findings of this study can help us gain a holistic understanding of the relationships between public emotional and behavioral responses in major US cities during COVID-19, as well as strengthen the practical significance of social media analytics for supporting the development of response strategies and activities.

## Methods

### Data

To efficiently isolate COVID-related tweets with geotagged data, we identify the subset of tweets collected in^20^ that have ‘geo’ or ‘user_location’ attributes (as opposed to the inferred locations identified by the study) within the 100 Metropolitan Statistical Areas (MSA’s) with the highest populations^21^. Geolocations with ‘city’ labels corresponding to subregions within each MSA were mapped to their associated MSA, and ‘geo’ attributes were given priority over ‘user_location’ attributes if both were available. Tweets were dated over the period from February 1st 2020 to May 1st 2020, and to reduce statistical noise, we only study MSA’s with an average of $\ge 500$ tweets per day over this period, which reduces the dataset to 49 cities. After initial inspection, we reduced the timeframe of study to begin February 26th, as all cities studied had a significant jump in tweet count on this day, with over 100,000 total daily tweets for the first time. This date also corresponds with the day when the CDC confirmed the first community transmission within the US, and so it is a key date in the evolution of the disease spread in the US. The final dataset consists of 12,670,890 tweets across the 49 cities, with a minimum count of 44,575 tweets (Grand Rapids, MI), a maximum count of 1,551,182 tweets (Washington, DC), and a median count of 170,234 tweets. The specific cities studied can be seen in Fig. 3.

Despite its high volume, Twitter data produces inherently noisy estimates in sentiment classification analyses due to its sparsity and high composition of non-standard characters^22,23^. To mitigate these issues as much as possible, we limit our analyses to aggregate trends in the polarity of tweets. Tweet sentiment was analyzed using the Amazon Comprehend API https://aws.amazon.com/comprehend/, which has been shown to outperform other off-the-shelf methods for correctly identifying tweets with positive or neutral sentiment, and vastly outperforms more naive methods^24^. In a period characterized by excess negative sentiment^25^, the ability to correctly identify tweets with positive and neutral sentiment is of the utmost importance, and so we opt for the Comprehend API due to this strength. We note some limitations of this approach in “Limitations” section. The API returns confidence scores (normalized to sum to 1) associated with the sentiment classifications {Positive, Neutral, Negative, Mixed} for the tweet being analyzed. However, as the method to obtain these scores is proprietary and confidential, we choose to simply utilize the sentiment of highest confidence as the classification for each tweet to maintain a simple interpretation of the measures we discuss. (Initial tests revealed that considering the confidence scores in weighted variants of our measures made little to no qualitative difference anyway.) The final dataset, consisting of Tweet ID (in compliance with the Twitter terms of use agreement) and primary sentiment classification for all 12,670,890 tweets is available at https://github.com/aleckirkley/US-COVID-tweets-with-sentiments-and-geolocations/.

For mobility data, we collect the daily city-level values for driving and walking from the Apple mobility trends reports https://www.apple.com/COVID19/mobility, which give the relative volume of Apple Maps route requests per city compared to a baseline volume on January 13th, 2020. Cities in this dataset are delineated by their corresponding greater metropolitan area, and so are geographically bounded to the same regions as the tweet data. We also utilize COVID case and death time series data in Fig. 3 to compare the correlations between sentiments and these pandemic statistics with the correlations we see between the sentiments and mobility measures. The values for confirmed daily cases and deaths in all counties within each MSA were aggregated from the JHU CSSE repository https://github.com/CSSEGISandData/COVID-19.

In order to assess how the dynamics of online geolocalized emotion were affected by major policy events in the early stages of the epidemic in the US, we identify three federal policies and one local policy to use as reference policy events. Due to the generally decentralized and casual approach to containment through policy interventions taken by the federal government during the early stages of the epidemic in the US^26–28^, it is difficult to clearly isolate key federal policy actions taken during this period. We thus identify the dates associated with three policy announcements during the time period studied that may reflect the general opinion about the state of the epidemic from the viewpoint of the federal government: (1) March 13, the date the US declared COVID-19 a national emergency; (2) March 29, the date President Donald Trump officially extended social distancing guidelines—discouraging nonessential workplace attendance and travel, eating at restaurants, and gatherings of more than ten people—through the end of April; (3) April 16, the date President Trump released a set of guidelines to states for reopening, based on the condition of the individual state. For local policy, we identify the dates each city instituted a shelter in place order, based on the dates of these policy announcements at the state level. We also record the dates that cities ended their local shelter in place orders (again based on state policies), if this occurred within the timeframe studied, and these are accounted for in the intervention analysis in Fig. 2.

### Online geolocalized emotion measures

Two measures are proposed in this section to analyze the online geolocalized emotion (OGE) in the 49 cities at the micro- and macro-level. The first is an intuitive measure capturing the average polarity of daily online sentiments in each city, which quantifies the positive or negative tendency of online sentiments toward COVID-19 in each geolocation group. The second measure analyzes the coherence of online sentiments across the 49 cities, and is used to examine the uniformity of online emotional responses at the country-level across time with respect to multiple subtopics. As the effects of COVID-19 are multifaceted, in addition to understanding the online emotional response based on all COVID-relevant geotagged content, people’s opinions about five important subtopics are also assessed: the Trump administration (abbr. “TA”), China (abbr. “China”), social distancing/quarantining (abbr. “distancing”), face masking (abbr. “mask”), and the economy (abbr. “economy”). We also denote tweets not restricted to any particular subtopic using the abbreviation “overall”. With the subtopics of interest chosen ahead of time, to identify keywords for each of these topics we look at the number of tweets related to all unique words in the full set of tweets, and identify high-frequency keyword sub-strings associated with each of the five topics. The keyword sub-strings identified with each topic are (all in lowercase, as were the cleaned tweets):

- “TA”: {“trump”,“pence”}
- “China”: {“china”,“chinese”,“wuhan”}
- “distancing”: {“quarantin”,“lockdown”,“social distanc”}
- “mask”: {“mask”,“ppe”}
- “economy”: {“econom”,“stock market”,“dow”,“unemploy”}

We choose only substrings that are found in the top $\sim 1000$ most frequent unique words, as well as only those we can unambiguously identify with a given subtopic (based on randomly sampling 100 tweets per substring for manual verification).

In the interest of interpretability, we use a very simple measure to quantify daily average OGE. Let $T^{topic}_C(t)$ be the total number of tweets on day *t* for city *C* mentioning the subtopic *topic*, with $P^{topic}_C(t)$, $M^{topic}_C(t)$ and $N^{topic}_C(t)$ the corresponding subset of these tweets associated with positive, neutral/mixed, and negative sentiment respectively such that $T^{topic}_C(t) = P^{topic}_C(t)+M^{topic}_C(t)+N^{topic}_C(t)$. To find the average polarity of tweets on day *t* related to a given subtopic *topic* in a city *C*, which we will call the “Geolocalized Mean Sentiment” (GMS) regarding *topic*, we assign a score of $+1$ to positive tweets, 0 to neutral tweets, and $-1$ to negative tweets, and take the average score. Mathematically, the GMS, *G*, is given by

1 $$\begin{aligned} G^{topic}_C(t) = \frac{P^{topic}_C(t)\times (+1)+M^{topic}_C(t)\times (0)+N^{topic}_C(t)\times (-1)}{T^{topic}_C(t)} =\frac{P^{topic}_C(t)-N^{topic}_C(t)}{T^{topic}_C(t)}. \end{aligned}$$

We can see that $G^{topic}_C(t)$ just amounts to the difference in the fraction of the tweets $T^{topic}_C(t)$ that are positive and the fraction that are negative, and is constrained to $[-1,1]$ with $-1$ indicating entirely negative tweets and $+1$ entirely positive tweets. Tweets of neutral and mixed sentiment are accounted for here in that the GMS is diminished in magnitude when they comprise a greater relative fraction of tweets for that day.

We define an additional online geolocalized emotion measure based on these GMS values, to quantify the amount of agreement in $G^{topic}_C(t)$ between all pairs of cities $\{C_1,C_2\}$ with respect to all five subtopics of interest simultaneously. First, we construct the GMS vector for each city *C*

2 $$\begin{aligned} \vec {G}_C(t)\equiv \{G^{distancing}_C(t),G^{China}_C(t),G^{Trump}_C(t),G^{economy}_C(t),G^{mask}_C(t)\}, \end{aligned}$$

which takes the form of a vector in ${\mathbb {R}}^5$. This construction associates each GMS topic with an orthogonal unit axis, which is consistent with our definition of these topics as independent topics of interest. We then define the angle $\theta _{C_1C_2}(t)$ between the vectors $\vec {G}_{C_1}(t)$ and $\vec {G}_{C_2}(t)$ (measured in degrees) as

3 $$\begin{aligned} \theta _{C_1C_2}(t) = \cos ^{-1}\left( \frac{\vec {G}_{C_1}(t)\cdot \vec {G}_{C_2}(t)}{\vert \vert \vec {G}_{C_1}(t)\vert \vert \; \vert \vert \vec {G}_{C_2}(t)\vert \vert }\right) , \end{aligned}$$

which will be $0^\circ$ when the cities $C_1$ and $C_2$ have collinear GMS vectors, $90^\circ$ when cities $C_1$ and $C_2$ have completely orthogonal GMS vectors, and $180^\circ$ when cities $C_1$ and $C_2$ have anti-parallel GMS vectors. Finally, we compute the “GMS coherence” $\phi (t)$ as

4 $$\begin{aligned} \phi (t) = \frac{2}{n_C(n_C-1)}\sum _{C_1\ne C_2}\theta _{C_1C_2}(t), \end{aligned}$$

where $n_C=49$ is the number of cities. This simply gives the “average angle” between the GMS vectors at time *t*, and is used as a proxy for the general agreement in GMS values over all cities at each time period. In particular, high values of Eq. (4) indicate low coherence in the GMS vectors across cities (as the average angle between them is high), and values near $0^\circ$ indicate high coherence, as the GMS vectors are all oriented similarly.

**Figure 1:**
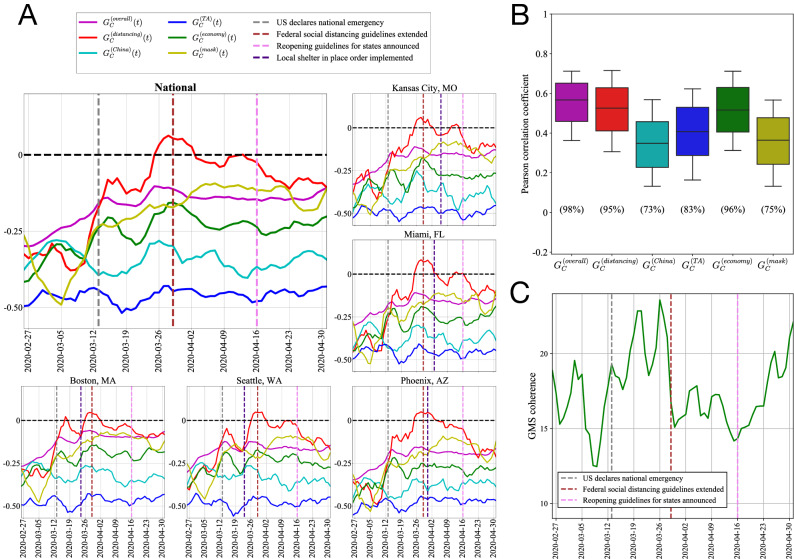
Universal trends in online geolocalized emotion towards COVID across US cities during the early stages of the epidemic. (**A**) Temporal trends for all GMS values considered (Eq. 1) over the time period studied (solid lines), for the entire dataset as well as for a selection of geographically dispersed cities, showing strikingly uniform temporal trends. Key policy events identified during this time period (dashed vertical lines) are shown for reference, and 7-day moving averages are displayed for the time series for clearer visualization. (**B**) Distribution of Pearson correlation coefficients between all pairs of (differenced) city-level GMS time series, for each GMS type, indicating high correlation in day-to-day GMS values across cities. Whiskers denote the 10th and 90th percentiles, and below each boxplot we display the percentage of all Pearson correlations for the corresponding GMS subtopic that were statistically significant at the 0.05 level. (**C**) GMS coherence (Eq. 4) over the time period studied (solid green line), indicating low variability in cities’ GMS vectors (Eq. 2) across time, with particularly consistent GMS values between the extension of distancing guidelines and the announcement of reopening procedures.

We note here that GMS (Eq. 1) and GMS coherence (Eq. 4) are used as simple metrics to capture online geolocalized emotion, but numerous similar constructions for OGE measures are possible. Assuming we’ve decided on a framework to classify tweet sentiment (a difficult problem in its own right^29^ that we will elaborate further on in “Limitations” section), the GMS could easily be constructed using the average of weighted sentiment scores output by this algorithm, rather than the more coarse approach assigning only values in $\{1,0,-1\}$. However, for this study we choose the GMS measure in Eq. (1) so as to not attempt to assign physical significance to the confidence scores output by the sentiment classifcation API, as these are constructed using an unknown proprietary method. Additionally, we should note that the coherence measure in Eq. (4) could be adapted to account for correlations between the subtopics by not treating them as orthogonal axes. For this alteration we could simply apply a coordinate transformation to the inner product in Eq. (3), making it the inverse covariance matrix between the bias vectors, and transform the norms in the denominator accordingly. (This procedure is more formally called transforming to “Mahalanobis space”^30^.) However, here we again opt for the simpler, more interpretable option of treating the subtopics as orthogonal unit axes, and stress that from experimentation the Mahalanobis transformation gives very qualitatively similar results for the coherence in Fig. 1C.

### Time series analyses

We use time series analysis—in particular intervention, correlation, and Granger causality analysis—to examine the relationships of GMS with policy development, offline mobility and infection data. For all time series analyses, we preprocess the series so that they are stationary to remove temporal trends. To do this, for any pair of series ${x_t,y_t}$ that are being compared, we use the following procedure

1. Perform an Augmented Dickey-Fuller (ADF) unit root test to check for stationarity of both series
2. If one or both series fail to reject the null-hypothesis (that there is a unit root in the series) at the 0.05 significance level, transform both series by taking differences $\{x_t,y_t\}\rightarrow \{x_t-x_{t-1},y_t-y_{t-1}\}$
3. Repeat 1 and 2 until null-hypothesis is rejected at the 0.05 significance level

Most series pairs only needed to be differenced once to satisfy these criteria, and in the worst case had to be differenced three times. For all time series analysis involving case or death data, both series are truncated to start when cases or deaths in the associated city become non-zero (which is necessary to pass the stationarity tests anyway). We also note that the application of variance-stabilizing transformations (in particular square-roots and logarithms) did not in general reduce the order of integration for the time series, and so we do not apply these to the data.

To assess whether or not each policy event had a significant impact on the GMS $G^{(topic)}_C(t)$, we use an ARMAX (Auto Regressive Moving Average with with eXplanatory variables) model, which can account for effects from the lagged dependent variable $G^{(topic)}_C(t)$ as well as exogenous categorical (binary) inputs $z_{kt}$ (policy events)^31^. In the ARMAX(*p*,*q*) process, the dependent GMS variable is modeled as

5 $$\begin{aligned} G^{(topic)}_C(t) = \sum _{i=1}^{p}\alpha _iG^{(topic)}_C(t-i)-\sum _{i=1}^{q}\gamma _i\epsilon _{t-i}+ \sum _{k=1}^{4}\beta _k z_{kt} +\epsilon _t, \end{aligned}$$

where *p* is the number of autoregressive terms, *q* is the number of moving average terms, *k* indexes the policy events, and $\epsilon _t$ is Gaussian white noise. For the declaration of national emergency ($k=1$ or $k=\text {national emergency declaration}$), extension of social distancing guidelines ($k=2$ or $k=\text {extension of distancing guidelines}$), and issuing of state reopening guidelines ($k=3$ or $k=\text {reopening guidelines announcement}$), we set $z_{kt}=1$ only on the date *t* of the announcement, and 0 for all other *t*, while for the local shelter in place orders ($k=4$ or $k=\text {local shelter in place order}$), we set $z_{kt}=1$ for the entire duration of the shelter in place order for each city and $z_{kt}=0$ otherwise. The ARMAX model amounts to a special case of the more general “transfer function” (or “dynamic regression”) models^32^, tools commonly used in econometric intervention analyses, which can be seen through rewriting Eq. (5) in the following form

6 $$\begin{aligned} G^{(topic)}_C(t) = \frac{1}{\alpha (\Delta )}\sum _{k=1}^{4}\beta _kz_{kt}+\frac{\gamma (\Delta )}{\alpha (\Delta )}\epsilon _t, \end{aligned}$$

where $\Delta$ is the differencing (or “backshift”) operator that transforms $x_t\rightarrow x_{t-1}$, and $\alpha (\Delta ) = 1-\sum _{i=1}^{p}\alpha _i\Delta ^i$ and $\gamma (\Delta ) = 1-\sum _{i=1}^{q}\gamma _i\Delta ^i$.

For each intervention analysis, we scan over the range of lags $(p,q)\in [0,7]\times [0,7]$ and pick the pair (*p*, *q*) with the lowest Bayesian Information Criterion (BIC), a model selection diagnostic based on the fit likelihood with a penalty for less parsimonious models^32^. Additionally, autocorrelation and partial autocorrelation functions of randomly sampled series were visually examined to verify the model fits, to ensure that significant autocorrelation lags in the GMS variables were properly accounted for in the ARMAX model. Using the ARMAX(*p*,*q*) process, we are able to see the nature and impact of a policy event $z_{kt}$ on $G^{(topic)}_C(t)$, after accounting for correlations from lagged values of this GMS variable and moving average terms, by analyzing the sign and statistical significance of the maximum likelihood estimates ${\hat{\beta }}_k$ inferred through this model. In particular, we look at the standardized *z*-score $Z^{(topic)}_k$ associated with each estimate ${\hat{\beta }}_k$ for the ARMAX model with dependent variable $G^{(topic)}_{C}(t)$

7 $$\begin{aligned} Z^{(topic)}_k = \frac{{\hat{\beta }}_k}{\sigma _{\beta _k}}, \end{aligned}$$

where $\sigma _{\beta _k}$ is the standard error of the estimate ${\hat{\beta }}_k$. Using $Z^{(topic)}_{k}$ allows us to assess both the sign and statistical significance of $\beta _k$ in a scale-independent manner. Similar to the pairwise analyses, the $G^{(topic)}_C(t)$ series were stationarized through differencing once (all that was needed to reject the ADF null at the 0.05 significance level for all cities) prior to intervention analysis.

For the correlation analyses in Figs. 1B and 3A, we perform the differencing procedure discussed at the beginning of this section to each pair of variables, and then compute their associated Pearson correlation coefficient. We also implement Granger (non-)causality testing in Fig. 3B to determine whether a given GMS variable, $G^{(topic)}_C(t)$, is theoretically able to provide additional statistically significant information for predicting the future values of each mobility variable, *M*, accounting for past values of *M*^33^. First, the optimal lag *p* for the univariate autoregression of *M* is determined by fitting

8 $$\begin{aligned} M_t = \sum _{i=1}^{p}\alpha _iM(t-i)+\epsilon _t \end{aligned}$$

(where again $\epsilon _t$ is a white noise process, and $\alpha$ are the autoregression coefficients) at a range of *p*’s and selecting the best fitting model using the associated BIC. Then, we add in the lagged values of $G^{(topic)}_C(t)$ and fit

9 $$\begin{aligned} M_t = \sum _{i=1}^{p}\alpha _iM(t-i)+\sum _{i=1}^{p}\omega _iG^{(topic)}_C(t-i)+\epsilon _t. \end{aligned}$$

Finally, we reject the null hypothesis that $G^{(topic)}_C$ does not Granger-cause *M* if any of the $\omega _i$ are determined to be significantly different than 0 through chi-squared testing^34^. GMS and mobility variables are differenced to the same level prior to testing, using the procedure outlined earlier.

## Results

### Common trends in online geolocalized emotional responses across US cities

As a first step in understanding the trends of OGE towards COVID-related topics across the major cities in the US during the early stages of the COVID epidemic, the daily geolocalized mean sentiment (GMS, Eq. 1) with respect to the five subtopics discussed in “Online geolocalized emotion measures” is plotted across time in Fig. 1A at the national level—computed based on all daily subtopic relevant tweets in the dataset, irrespective of location—and for five geographically dispersed example cities. We also show the dates of the policy events identified in “Data” for reference, and values are plotted using a weekly moving average to smooth out fluctuations for easier visualization. The first pattern we can observe is the strong similarity between the trends in the national GMS and the GMS in the five example cities shown, for all subtopics. This suggests that, at the city level, there is little heterogeneity in COVID OGE trends geographically, in contrast to policy response across local governments for which there has been a high level of heterogeneity^35,36^.

Looking at the trends in Fig. 1A in more detail, we can see in general that GMS is negative for all subtopics, and there is a similar ordering in the GMS values for the subtopics and overall GMS over time, with “distancing” typically garnering the most positive GMS and “TA” typically garnering the most negative GMS. We also see relative stability in “TA” and “China” GMS values, while “distancing”, “economy”, “mask”, and overall GMS values tend to increase over the period studied. There are relatively strong fluctuations in the trends for many of the GMS series near the date of President Trump’s extension of social distancing guidelines, particularly in “distancing”, which even reaches above zero during this period for all examples shown. This reflects a general sense of positivity about social distancing-related behaviors surrounding this announcement, perhaps indicating people’s commitment to, or resilience regarding, continued distancing.

To compliment our qualitative visual analysis in Fig. 1A–C we investigate more quantitatively whether or not there is a strong correlation in the GMS values across these cities. Shown in Fig. 1B are the distributions of Pearson correlation coefficients between city-level GMS values across all pairs of cities, for overall GMS and all subtopics of GMS. Series were stationarized through differencing once prior to analysis, and so the correlations we see are actually between day-to-day changes in GMS values. Also shown in Fig. 1B below each boxplot is the corresponding percentage of all Pearson correlations that were statistically significant at the 0.05 level for that subtopic. We can see that the Pearson correlations across all city pairs are relatively high for overall GMS and all GMS subtopics, indicating that not only the temporal trends are similar between these GMS variables, but the daily fluctuations are also highly correlated. This is reflected in the high percentage of significant correlations as well.

In Fig. 1C we assess a different dimension to this homogeneity in GMS, aggregating GMS with respect to all subtopics (excluding overall GMS) into temporal vectors and looking at the similarity in these vectors over time through the coherence measure in Eq. (4). We also plot a moving average, this time a three-day moving average, to more easily visualize trends. We can see that the GMS coherence over time is pretty stable, fluctuating between $\approx 13^\circ$ and $\approx 24^\circ$ (relative to a maximum value of $180^{\circ }$), and maintaining relatively low values, indicating high similarity in the GMS vectors over time, consistent with the findings in the other two panels. However, we also observe some disturbances in the pattern occurring around federal policy event dates. More specifically, we see an increase in GMS vector similarity (through a declining coherence measure) near the extension of social distancing guidelines, and a transition in the trend around the state reopening guidelines announcement from relatively unchanging to increasing. Noting the generally high similarity in the OGE dynamics across cities seen in Fig. 1A,B, as well as the low values of Eq. (4) in Fig. 1C, these disturbances provide initial evidence that we still see heterogeneity in city-level OGE towards COVID-related topics, but it manifests itself in the direct influence of policy events, which is a much more subtle factor to address. We perform the analysis necessary to address these effects in the next section.

### Sensitivity to federal and local policies

**Figure 2:**
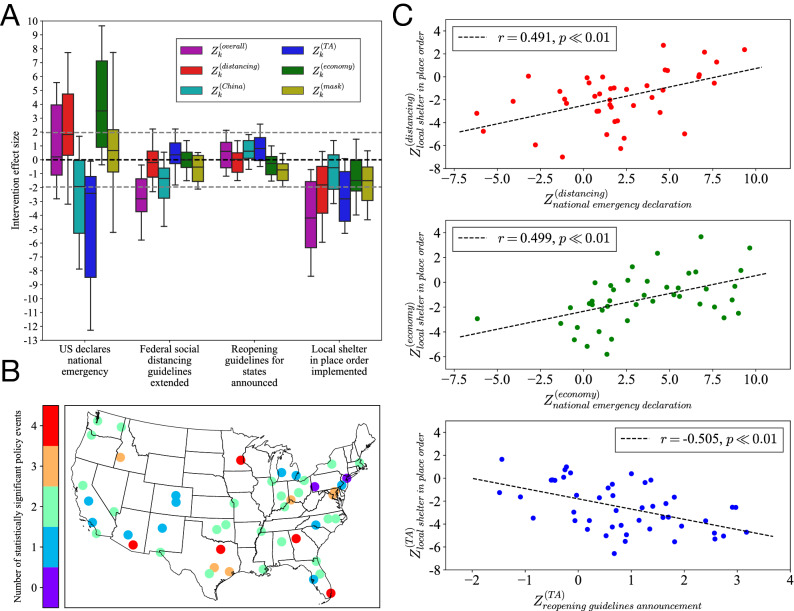
Effects of major federal and local policies on online geolocalized emotion. (**A**) Policy effect sizes $Z^{(topic)}_k$ (Eq. 7) for all GMS values *topic* and policy events *k*, displaying high variation across cities in response to the national emergency declaration and local shelter in place orders, and lower sensitivity to the announcement of federal guidelines for social distancing extension and state reopening. Also shown are gray horizontal lines indicating the positions at which effect sizes are statistically significant at the 0.05 level with respect to a standard normal distribution. (**B**) Number of statistically significant responses in overall GMS $Z^{(overall)}_k$ (at the 0.05 level) to events $k\in [0,4]$, for all cities, indicating the general sensitivity of cities to the policies enacted during the period of study. (**C**) Effect sizes for various policy events on $Z^{(distancing)}$ (top), $Z^{(economy)}$ (middle), and $Z^{(TA)}$ (bottom), showing high Pearson correlations *r* between GMS responses regarding both displayed policy events for each topic. Extreme outliers with $\vert Z^{(topic)}_k \vert>>10$ are omitted from the OLS regressions, and the associated *p*-values are displayed alongside the Pearson correlation coefficients *r*.

To assess the extent to which each major policy event (detailed in “Data”) has an effect on the dynamics of online geolocalized emotion towards COVID-related topics in each city, we perform the intervention analysis discussed in “Time series analyses” for each subtopic GMS $G_C^{(topic)}$ and each city *C*, extracting the effect sizes $Z_k^{(topic)}$ in Eq. (7). In Fig. 2A we show the distribution of these intervention effect sizes $Z_k^{(topic)}$ regarding each subtopic for all cities across the four events identified for analysis. We observe generally strong effect sizes for the declaration of national emergency and local shelter in place orders, while the extension of distancing guidelines and announcement of state reopening guidelines have relatively weak values of $Z_{k}^{(topic)}$. The declaration of national emergency appears to have a very mixed effect on GMS values, with the distributions for $Z^{(China)}_{1}$ and $Z^{(TA)}_{1}$ displaying a strong tendency towards negative values, and the other variables showing a tendency towards positive values. Around $50\%$ of cities have “China” and “TA” subtopic GMS dynamics that are negatively affected by the declaration of national emergency to a statistically significant extent, indicating that these two topics were associated with a high level of negative sentiment as a result of the declaration. This is consistent with the high level of anti-Chinese sentiment observed during the early stages of the epidemic^37^, and expressions of anger on social media towards both US leadership and China^38^, although here we gain a more nuanced understanding of the effect a specific event has on these responses at a local level. We can also see generally negative intervention effect sizes connected with the implementation of local shelter in place orders, particularly on overall, “distancing” and “TA” GMS values, perhaps reflecting the anger and frustration associated with quarantining^39^. We compliment this illustrative analysis of intervention effect sizes in Fig. 2B with a visualization displaying the sensitivity of each city studied to the policy events, as measured by the total number of policies by which the city’s overall GMS values were affected to a statistically significant extent (at the 0.05 level). Here we see a moderate geographic trend, with cities in the southeastern US and Texas having generally more significant policy responses, and cities in the northeastern and southwestern US having generally fewer significant policy responses. However, we still see variability within each region, and so these responses are not necessarily well localized in space.

The sensitivity of cities to policy events is further investigated in Fig. 2C, where we plot the intervention effect sizes $Z^{(topic)}_K$ for different policy events *k* for the same city on each axis, with the three panels showing different subtopics *topic*. We perform three OLS linear regressions, one for each of the three pairs of variables, and determine through low autocorrelation of approximately normally distributed residuals as well as low *p*-values in all cases that these linear fits are appropriate models for the data. We observe in the top panel of Fig. 2C that cities responding more negatively about distancing after the national emergency declaration also tend to respond more negatively about distancing after their local shelter in place orders, and likewise for cities responding positively. We also see the same trend for the GMS responses relevant to the economy in the middle panel. The bottom panel shows that when comparing responses to the reopening guidelines announcement and responses to local shelter in place orders, we actually see the opposite effect, at least regarding “TA” biases. The negative correlation we see in this panel may reflect the different natures of the local shelter in place orders and the reopening guidelines announcement: for cities that respond negatively to local shelter in place orders, the announcement of reopening guidelines may be seen as a statement of optimism. On the other hand, for cities that respond positively to local shelter in place orders, the announcement of reopening guidelines may seem premature. However, making these determinations conclusively requires a more contextualized analysis with the aggregation of data from different sources. The results in Fig. 2 altogether indicate that some cities tend to be more sensitive to federal and local policy in their COVID OGE dynamics than others, and comparison with the results in Fig. 1 suggests that the heterogeneity in city-level OGE dynamics is better reflected by cities’ GMS responses to policy events rather than the overall observed trends and day-to-day correlations in GMS. Keeping in mind these observations about the manifestation of OGE at the city-level, we transition in the next section to analyzing its connection with local offline factors such as epidemic indicators and human mobility.

### High associations between online emotional responses and offline mobility

**Figure 3:**
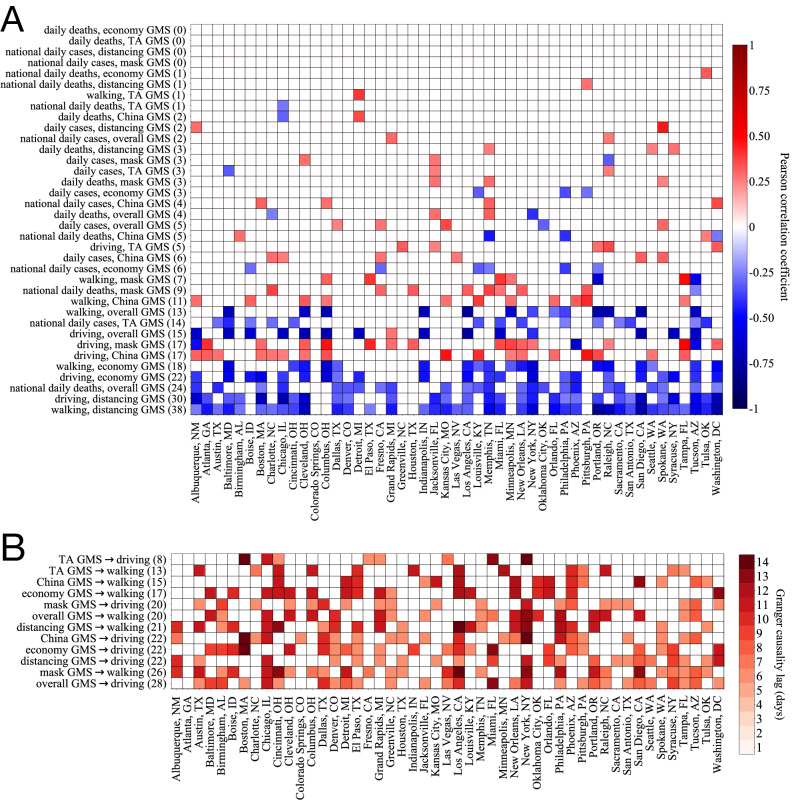
Association between online geolocalized emotion and offline factors. (**A**) Pearson correlations between (stationarized) GMS values and offline indicators, showing high correlation between GMS and mobility measures, but weaker correlations with epidemic measures. Only correlations that are statistically significant at the 0.05 level are shaded, and rows are ordered top to bottom by the number of cities with a significant correlation between the corresponding measures, which is shown in parenthesis alongside each pair of variables. (**B**) Inferred statistically significant Granger causality lags (Eq. 9) for mobility measures with lagged GMS variables, indicating that prediction of future mobility in cities is consistently aided through the information contained in the GMS values in many cities. Again, only lags for causality tests significant at the 0.05 level are shaded, and rows are ordered by number of cities with statistically significant causalities, which is labelled in parenthesis alongside variable pairs.

As a final investigation into the dynamics of online geolocalized emotion regarding COVID in cities, we look at its association with epidemic indicators (daily cases and deaths) and mobility measures (relative walking and driving volume). Further explanation of the epidemic and mobility datasets integrated into our analysis can be found in “Data”. In Fig. 3A, we plot the Pearson correlation coefficient for all pairs of offline and GMS variables to determine the strength and nature of the unlagged temporal correlation between these quantities within each city. Each pair of variables was differenced until both were stationary by using the procedure discussed in “Time series analyses”, which is crucial for eliminating the confounding temporal trends in all the variables studied. It is reasonable to guess that epidemic indicators may have instantaneous daily correlations with OGE: the abundance of online publicly available data and constant national and local media coverage of case and death statistics results in high, instant exposure to epidemic updates, the psychological effects from which have been discussed at length in current research^40,41^. However, we can see from Fig. 3A that epidemic statistics actually have very little correlation with GMS values. Only two pairs of variables involving epidemic indicators have statistically significant correlations in more than ten cities, while nine pairs involving mobility measures do. We can also see that among these generally low correlations, national daily cases and deaths have significant correlations with GMS in substantially more cities than local daily cases and deaths. In general, the significant correlations between case/death data and GMS values tend to be negative, indicating that emotional responses have a greater negative tendency as epidemic indicators grow more rapidly.

As opposed to epidemic indicators, we find that mobility measures are consistently highly correlated with many of the GMS subtopic measures. In particular, we see consistently strong and statistically significant negative correlations between mobility and $G^{(distancing)}_C$ as well as $G^{(economy)}_{C}$ in many cities, a point which we elaborate on in “Integrating online and offline data for crisis management: opportunities and challenges”. We also see strong correlations between mobility measures and $G^{(overall)}_C$ as well as $G^{(China)}_C$, though in fewer cities, and $G^{(TA)}$ and $G^{(mask)}$ appear to have much weaker associations with mobility measures. An interesting aspect of these correlations is that they are actually different in sign among various GMS subtopics: “distancing”, “economy”, and overall GMS values tend to have negative correlations with mobility measures, while “China”, “mask”, and “TA” GMS values tend to have positive correlations with mobility measures. Based on these mixed relationships between GMS subtopics and offline mobility, the impact of mobility on OGE regarding each subtopic individually is unclear, though we do know that there is a consistent statistical association between these quantities. The underlying psychological reasons for these connections between OGE and mobility can be investigated by future studies. However, for practical risk management, if we can use this online geolocalized emotion to predict future mobility patterns, this can aid in effective intervention plans. We thus look at a more general formulation of statistical association in Fig. 3B, assessing whether or not past GMS values can theoretically provide statistically relevant information about future mobility.

In Fig. 3B we show the optimal Granger causality lag for all pairs {GMS variable, mobility variable} that have a statistically significant Granger-causal relationship (details given in “Time series analyses”). Interpreting Granger causality as an indicator of theoretical predictability, we can see that GMS can consistently be used to aid in the prediction of future mobility values for most cities, and that frequently this prediction is possible at lags of a week or greater. We also note that the cities with the largest populations—in particular New York, Los Angeles, and Chicago—have significant Granger-causality across nearly all pairs of variables, with longer lag times that tend towards two weeks due to long-range temporal autocorrelations in the mobility values in these areas. Investigating the causes of this peculiar pattern, however, is outside the scope of this work. These results, along the correlations seen in Fig. 1A, suggest that there is a high statistical association between OGE and mobility at the daily level, and that the former can be effectively used to aid prediction of the latter with substantial foresight.

## Discussion

### Integrating online and offline data for crisis management: opportunities and challenges

In this study, we find that federal and local policies influence online geolocalized emotion towards COVID-19 in a variable manner across cities. Additionally, offline local mobility—an important indicator for assessing the effectiveness of social distancing policies—is found to be correlated with OGE in many cities. Based on Granger causality analysis, we further find that OGE can be used to aid in forecasting future offline local mobility. These interdependent relationships suggest a feedback loop between policy development, online sentiment, and offline mobility during the pandemic. Understanding and utilizing this feedback loop can facilitate the design and implementation of effective public health and disaster relief policy.

Due to the broad impacts of the pandemic, all analyses were conducted using the overall OGE as well as OGE regarding five sub-topics relevant to COVID-19. Despite the wide range of subtopics ranging from the economy to masking, strong statistical commonalities in overall OGE trends and those regarding subtopics were discovered across major US cities. This could be due to characteristics shared by urban populations active on social media that may contribute to similar trends of online sentiment during crises. However, we also find that the online geolocalized emotion towards crisis relief policies during COVID varied across cities. We analysed the intervention effects of three social distancing policies on the OGE across major US cities, finding that local shelter in place orders had strong immediate effects on online emotional response across cities, while the two federal social distancing guidelines did not contribute to significant fluctuations in OGE. This indicates that, in aggregate, the public may have a stronger emotional reaction to localized policy that has a direct impact on their behavioral responses, rather than federal guidelines suggesting behavioral changes. Since offline local mobility is associated with online emotional responses, these results can further indicate that mere suggestions by federal authorities may not solicit necessary behavioral compliance during crises, and that local enforcement of guidelines could be more effective.

It is also found that cities tend to have consistent collective emotional responses towards policies with similar objectives during the COVID crisis. For example, cities with more negative responses about the Trump administration immediately following local shelter in place orders generally had a more positive response to the administration following the announcement of reopening guidelines. On the other hand, cities with more positive responses about the Trump administration immediately following local shelter in place orders generally had a more negative response to the administration following the announcement of reopening guidelines. In this case, the first policy implies greater personal restrictions, while the latter policy relaxes these restrictions, and so the OGE patterns observed may reflect local beliefs or attitudes towards personal freedoms. Uncovering correlations between OGE responses across different policies may thus inform local policy makers about more general patterns in public opinion that may be more difficult to capture through traditional methods such as polling. In a more immediate context, federal and local authorities can consider to incorporate emotional assessment measures through social media for understanding public attitudes towards policies during the early stages of a crisis to develop the delivery plan for follow-up policies and provide effective spatially targeted interventions. Of course, such monitoring comes with ethical concerns as well, which we will discuss.

We also observe a moderate geographic trend in policy sensitivity, as cities in the southeastern US and Texas generally have more policy responses (in terms of overall COVID-related OGE) that are statistically significant than do cities in the northeastern and western US. Identifying such regional patterns in emotional responses during crises can allow for large-scale coordination of local governments to produce interventions, rather than relying solely on city-level policies for crisis mitigation.

In addition to informing policy development based on OGE regarding existing policies, we can use the connections between OGE and offline factors to assess the local situation during crises, which in turn can inform policy. Human mobility can serve as a primary indicator for the efficacy of crisis relief measures, particularly in the context of evaluating social distancing policies and mobility restrictions to reduce the spread of a pandemic. For this reason, understanding what factors drive human mobility patterns is of the utmost importance. We find that fluctuations in OGE are correlated with fluctuations in the relative volume of driving and walking across many cities. There are particularly consistent significant negative correlations between mobility levels and OGE regarding both distancing and the economy. Intuitively, mobility and distancing should be closely related—the more positive one is about social distancing/quarantining the more likely they are to stay home and decrease their overall mobility. However, the connection with sentiments about the economy is less clear, but it may relate to panic-buying or other activities driven by economic uncertainty. In terms of health policy, the former result may suggest the encouragement of social distancing through information campaigns with a more positive framing, pointing out the benefits of the practice as well as highlighting activities to do at home. The latter finding may suggest an increased emphasis on economic positivity when framing health policies, if indeed this can effectively reduce the drive for individuals to mobilize.

OGE is correlated with current mobility patterns, but as we have shown, it can also be used to aid in the prediction of future mobility patterns. Developing accurate predictions of future mobility, as well as identifying which crisis-relevant factors influence these predictions, could be used to develop preemptive measures to mitigate future risk, even if these measures are not solely based on the current local situation. On the other hand, arguably the most relevant statistics for assessing the state of a pandemic in a region are the daily changes in cases and deaths, although our analysis suggests that these statistics are very weakly correlated with OGE. This calls for careful evaluation about using social media data for crisis management—online emotional responses may not accurately reflect the actual state of the crisis from a scientific point of view.

Aside from the practical implications of integrating online and offline data for emergency response—particularly in the case of the current pandemic as in this study—there are also ethical concerns that call for attention. Even though linking large-scale user-generated data on social media with offline mobility data only requires geo-tags, rather than direct personal identifiers, it is important to take the appropriate measures in data collection from social media for protecting the privacy of personal information. This requires the authorities and companies who have access to the data sources to employ an ethics-driven approach to use, manage and store the data only for public health and relief purposes. It is also important to be aware of sample representativeness while using social media data or mobile phone data for informing policies, as the digital divide and unequal access to digital technologies excludes certain populations from these data samples. We elaborate on this issue in the context of our study in the next section.

### Limitations

It is important to additionally note some key limitations of our approach, particularly regarding the data aggregation and sentiment analysis, as applications of our methodology for future studies may be improved through refining these aspects.

Firstly, it is critical to assess the representativeness of the Tweet sample used in the analysis, as this can have a clear impact on outcomes for public health interventions^42^. One major limitation of using geolocalized social media data is that it may be biased towards certain demographics— such as young adults and women^43^—due to the deliberate inclusion of the geographic information along with the tweet^44^. As geolocation is fundamental to our approach, one possible way to mitigate this bias is to sample tweets both with and without explicit geolocation metadata, and infer the geolocations for those without geolocation metadata^45^. However, this approach may introduce lots of uncertainty due to the predictions of the geolocations, and so the costs and benefits must be balanced for such an approach to be utilized in practice. Another critical issue to consider—particularly given that geolocated tweets tend to over-represent urban populations^46^—is the divide in crisis response between urban and rural areas. Due to various factors including local news coverage^47^ and access to healthcare facilities^48^, we have seen a significant discrepancy in pandemic response between urban and rural populations^49^. In this sense, the universality and other findings of this study could be restricted to urban populations, and additional future work is necessary to assess online geolocalized emotion and its correlations with offline factors in rural areas. Finally, only tweets with COVID-19 related hashtags and keywords are used in our dataset, rather than a sample of tweets from the entire Twitter streaming API. Tracking all COVID-related content on Twitter is difficult due to the dynamic nature of the relevant content, and our dataset is derived from one in which 803 manually chosen trending keywords and hashtags were used to extract tweets^20^, which may result in a lack of full coverage of all COVID-19 relevant content on Twitter. Furthermore, future studies can also consider integrating online and offline data by assessing online geolocalized emotion in general, based on a random subset of *all* tweets rather than just those that explicitly mention COVID-19 related keywords. Unfortunately, any social media-based findings will exclude populations without access to the associated technologies (computers, smartphones, etc), and this limitation is pervasive in any of the suggested alternative data aggregation methods presented here.

In addition to the data collected, the methods by which sentiment is categorized for the tweets of interest also present potential limitations. Sentiment classification techniques can be broadly categorized into techniques that are (1) machine learning-based and (2) lexicon-based, both having their own advantages and disadvantages^50^. Machine learning methods may outperform the latter in terms of classification accuracy on real-world text data when labelled training data is available, as they are able to automatically extract sentiment-relevant information from text rather than relying on human judgement. However, machine learning methods tend to have poor translational capability across domains, and so it can be argued that lexicon-based methods are more appropriate for some tasks where domain specific training data is not available in large quantities due to their interpretability and simpler manipulation. The current study focuses on a broad array of subtopics of interest relevant to COVID-19, and there is a scarcity of high quality COVID-specific manually labelled training data. Consequently, an off-the-shelf method (Amazon Comprehend) was preferred over manually trained machine learning algorithms or lexicon-based approaches due to its broad domain applicability, computational efficiency, and accuracy on a small manually labelled subset of the data. However, this potential improvement in classification accuracy and computational efficiency comes at the price of interpretability, although this is the case with most highly parameterized supervised learning approaches, proprietary or not. Finally, another limitation that can be considered to improve future studies pertains to assessing aspect-based sentiment using tweets. Since the dynamics of online geolocalized emotion are studied with respect to the subtopics of COVID, it is possible that *aspect-based*^51^ sentiment classification—categorizing subtopics within a tweet and determining sentiment towards these—is more appropriate for the present study than sentiment classification of the overall tweet mentioning a given subtopic. However, due to the brevity of tweets (the average length is around 30 characters, with a maximum possible 280), tweet-level sentiment classification may be a good approximation to the results from aspect-based approaches, as tweets cannot possibly span a very broad range of topics. It is also possible that many systematic biases—even severe ones—in sentiment classifications may not significantly affect the findings of this study, as long as they are consistent across times and locations. As temporal fluctuations of (and correlations between) these sentiments are more relevant than the absolute values of the sentiments themselves, time- and location-independent biases in geolocalized mean sentiment will not influence the results.

## Conclusions and future work

In this study, we examine online geolocalized emotional (OGE) responses towards COVID and five related subtopics across 49 US cities from Feb 26th to May 1st 2020 using a dataset of around 13 million tweets with geolocation attributes. We assess the temporal dynamics of OGE in these cities through a few sentiment-derived measures, as well as analyze the associations of OGE dynamics with critical COVID-relevant policy events, offline mobility, and epidemic measures. The key findings of this project related to our original research questions are: (1) There is a universal temporal trend in OGE towards COVID across US cities, with high day-to-day correlations and consistent relative negativity in sentiment across the COVID subtopics; (2) OGE across cities is sensitive to major federal policy announcements and local shelter in place orders, and some cities are much more consistently sensitive to policy events than others; (3) OGE is highly correlated with mobility but not with epidemic measures, and OGE has a high predictive capability for future mobility. The findings of this study help us to understand the city-level manifestation of public online emotional responses during the COVID crisis in the US, and how this online collective emotion connects with offline factors such as policy, epidemic measures, and human mobility.

There is a plethora of possible future work extending the ideas presented in this study. One clear avenue for future work is the extension of the timeframe studied to incorporate data up to the present day, and the application of these methods to OGE dynamics in cities worldwide. Another important extension is to incorporate a more refined set of sentiment classifications—for example including classifications for fear or anger as subsets of negative sentiment—and constructing new OGE measures based on these categories. Additionally, our framework can be adapted to examine other important aspects of public behavioral responses (such as purchasing behavior) or demographic factors (such as socioeconomic status), and how these connect with online geolocalized emotion during crises. Finally, the practical application of predicting future values of mobility using OGE is a critical avenue for study that builds off of this project, which can be used in conjunction with existing studies assessing the impact of human mobility on epidemic spread to make informed policy decisions^52–54^.

## Data Availability

Tweet IDs and their inferred sentiments have been made openly available online by the authors at https://github.com/aleckirkley/US-COVID-tweets-with-sentiments-and-geolocations/. Mobility data is available at https://www.apple.com/COVID19/mobility, and epidemic statistics can be downloaded from https://github.com/CSSEGISandData/COVID-19.
